# Misuse of Antipyretic Amid Fear of COVID-19 Vaccine

**DOI:** 10.31729/jnma.6262

**Published:** 2022-03-31

**Authors:** Arun Gautam, Urza Bhattarai

**Affiliations:** 1 Galkot Primary Health Care Centre, Baglung, Nepal; 2 All India Institute of Medical Sciences, New Delhi, India

**Keywords:** *antipyretics*, *COVID-19 vaccine*, *drug misuse*, *vaccine immunogenicity*

## Abstract

The COVID-19 vaccination campaign is going on in Nepal through different phases of immunisation. It has been observed that people are misusing antipyretics and analgesics with the fear of adverse events following immunisation. The possibility of antipyretics and analgesics blunting the antibody response of the human body can be a potential cause for lower immune response and thus a reason for lower efficacy of the vaccine. Prophylactic use of over-the-counter analgesics and antipyretics is to be discouraged until the data for or against its use is available.

## INTRODUCTION

The COVID-19 pandemic started in the year 2020. Many vaccines have been developed against this disease. Few have been approved for emergency use and few are still under early clinical trials.^1^ COVID-19 vaccination campaign is going on in Nepal since 27^th^ January, 2021 through different phases of immunization.^2^ The vaccine: ChAdOx1 nCoV-19 that is being used in Nepal is approved by World Health Organisation (WHO) for its emergency use.^3^ This Vaccine is found to have various adverse events. Adverse events like feverish feel, fever, headache, injection site pain, weakness, joint pain, and myalgia are common while dizziness, abdominal pain, and lymphadenopathy are uncommon.^4^ It was found that those who took the vaccine had used antipyretics or analgesics or both, as prophylaxis.

## THE GROUND SCENARIO

As community-level health care workers who are directly involved in a vaccination campaign, we came across many concerns regarding this vaccine in the public. The message of the vaccination campaign was delivered through media like FM, miking, posters and pamphlets. Community-level health care workers and the female community health volunteers (FCHVs), local level government bodies were actively mobilised to convey information regarding the date and site of the vaccination campaign and the importance of vaccine use during the pandemic. The trained rapid response Adverse events following immunisation (AEFI) management team was formed at each rural municipality/municipality level. Elderly and differently-abled citizens were provided transport facilities for a vaccination with support from local government bodies.

There was a good participation of individuals in the vaccination campaign. We found mixed perceptions of the participants who came for vaccination. On one side there was enthusiasm on the availability of the awaited Covid vaccine at a community level while on the other side there were concerns regarding the safety and efficacy of this vaccine. As the vaccine use was already approved for emergency use by WHO, we were confident in reassuring the participants. Along with hesitancy, we also found fear regarding the adverse events that may follow vaccination. We observed all of the participants for half an hour postvaccination in the health centres and provided them with information on possible adverse events that can appear. All the participants of the vaccination campaign were provided the contact numbers of health workers and medical doctors for any queries and for seeking help for any difficulties following immunisation. We found the enthusiasm of participants in sharing their post-vaccination experiences among themselves and with the health care workers.

## THE ANTIPYRETICS MISUSE

Few of the participants who took the vaccine were found to have used antipyretics or analgesics or both, as prophylaxis. The majority took Paracetamol and Ibuprofen within a few hours of vaccination because of the fear of adverse events like fever, headache, and body ache. There are numerous ongoing awareness programs regarding vaccine use, its safety, and possible minor adverse reactions. The possible over-the-counter use of antipyretics prophylactically by the public could be in response to the fear of minor adverse events that have been disseminated during awareness campaigns. But at the same time, it suggests that we failed to convey awareness regarding the misuse of prophylactic over-the-counter antipyretics and analgesics. As this is a newly launched vaccine, the effect of such drugs on antibody response is yet to be determined.^5^ The use of antipyretics has been shown to blunt the immune response of the body towards the vaccine, while few studies have also mentioned no significant effect of such medications on the efficacy.^6^ Moreover, the World Health Organization, in its guidelines, has advised against their use immediately before or after vaccination.^7^ This vaccine is found to have an efficacy of 70.4% (95.8% CI of 54.8 to 80.6%) occurring more than 14 days after the second dose.^1^ Although one study mentions that the immunogenicity of this vaccine is not affected by the prophylactic use of Paracetamol, there is a lack of data regarding the same.^8^ Also, the effects of NSAIDs like ibuprofen that are commonly used in developing countries like Nepal, are not studied. The possibility of antipyretics and analgesics blunting the antibody response of the human body can be a potential cause for lower immune response and thus a reason for lower efficacy of the vaccine.

## WAYS FORWARD

A large number of people around the world could be using over-the-counter analgesics and antipyretics before or immediately after COVID-19 immunisation like in our community. The study regarding the effect of such drugs on the immunogenicity of these vaccines is lacking. Prophylactic use of over-the-counter analgesics and antipyretics should be discouraged until the data for or against its use is available.
